# Switching From Intravitreal Ranibizumab (Lucentis) to Biosimilar Ranibizumab (Ongavia) Injections: Insights From a Tertiary Care Eye Centre

**DOI:** 10.7759/cureus.101850

**Published:** 2026-01-19

**Authors:** Evdokia Sourla, Naima Zaheer, Randhir Chavan

**Affiliations:** 1 Ophthalmology, Birmingham & Midland Eye Centre, Birmingham, GBR

**Keywords:** anti-vascular endothelial growth factor (anti-vegf), clinical audit, diabetic macular oedema, diabetic retinopathy, intravitreal injections, wet-aged related macular degeneration

## Abstract

Background: Ranibizumab has been widely used to treat retinal conditions such as wet age-related macular degeneration, diabetic macular oedema, and macular oedema secondary to retinal vein occlusions. In 2022, the Medicines and Healthcare products Regulatory Agency (MHRA) approved Ongavia, the first biosimilar of ranibizumab. Ongavia offers comparable efficacy and safety, with a similar side effect profile to the original medication, while being more cost-effective.

Method: The study was conducted in the medical retina department of a tertiary care eye hospital. Data were collected from patients undergoing treatment with intravitreal ranibizumab (Lucentis) who were switched to the ranibizumab biosimilar, Ongavia, between November 2023 and February 2024. This study included two components. The first was a cross-sectional observational survey, in which patients being switched to Ongavia completed a satisfaction questionnaire regarding the information leaflet received and the discussions held about the switch. The second component was a retrospective review to assess the effectiveness and safety of ranibizumab biosimilar Ongavia in patients with wet AMD. After two Ongavia injections, the treatment interval for the next injection was reviewed and compared with the treatment interval with ranibizumab. Similarly, OCT scans before and after the switch were reviewed and compared. Any adverse effects documented in the clinical notes were recorded.

Results: Out of 121 eyes, 92 eyes (76%) were switched to biosimilar ranibizumab (Ongavia) injections, while 18 eyes (15%) continued receiving ranibizumab injections. Patient satisfaction with the information process was 100%. Eighty-seven eyes of patients with wet AMD received more than two Ongavia injections as per the treat-and-extend protocol. Out of these 87 eyes, in 64 eyes (73.5%), injection intervals were either maintained or extended. The treatment interval was reduced in seven eyes (8%), and nine eyes (10.3%) were switched to a different anti-VEGF medication. No safety concerns were identified with biosimilar ranibizumab.

Conclusions: Patient satisfaction with the information process was high, likely due to active involvement in treatment decision-making. The results indicate that the clinical effectiveness of ranibizumab biosimilar Ongavia is comparable to ranibizumab and that it has a similar safety profile. Ongavia could serve as a cost-effective alternative, reducing healthcare system costs while maintaining high standards of patient care.

## Introduction

Ranibizumab (Lucentis; Genentech) is a widely used anti-vascular endothelial growth factor (anti-VEGF) therapy, approved by the National Institute for Health and Care Excellence (NICE) and the U.S. Food and Drug Administration (FDA) for the treatment of wet age-related macular degeneration (wet AMD), macular oedema secondary to diabetes, retinal vein occlusions (RVO), and myopic choroidal neovascularisation [1]. Ranibizumab is a humanised monoclonal anti-VEGF fragment administered intravitreally at a dose of 0.5 mg. It works by targeting vascular permeability, angiogenesis, and inflammatory responses through inhibition of VEGF signalling [2]. The efficacy of intravitreal ranibizumab in treating wet AMD and diabetic macular oedema (DMO) is well established, with numerous supporting studies [3,4]. However, it is associated with a significant financial burden on healthcare systems.

Biosimilar drugs present an opportunity to reduce this financial burden and offer substantial benefits to both patients and healthcare systems by lowering costs, as biosimilars are priced lower than their originator counterparts. According to the World Health Organisation (WHO), biosimilars are biological products that are highly similar to an already licensed reference product in terms of quality and clinical and non-clinical evaluation. A biosimilar must demonstrate comparable pharmacokinetics, pharmacodynamics, safety, and efficacy to the innovator biologic [5].

Several ranibizumab biosimilars are currently under study and are at various stages of approval. In 2022, the Medicines and Healthcare products Regulatory Agency (MHRA) approved Ongavia (Bioeq AG, Switzerland/Teva Pharmaceuticals, Israel), a biosimilar of ranibizumab, as an anti-VEGF injection for treating wet AMD and DMO [6,7]. Ongavia is the first biosimilar of ranibizumab to receive approval, and the United Kingdom became the first country in Europe to begin its use [7]. The purpose of this study was to assess switching from ranibizumab to biosimilar ranibizumab.

## Materials and methods

Background

Starting in November 2023, all patients in our trust who were receiving intravitreal ranibizumab for DMO, wet AMD, and RVO were switched to the biosimilar ranibizumab, Ongavia, in accordance with the trust’s new policy. This change in practice was approved by the Trust Governance Team and the Drug and Therapeutic Committee (DTC).

According to the departmental protocol, an information leaflet describing the biosimilar ranibizumab Ongavia was sent to each patient’s home address along with details of their next intravitreal injection appointment. On the day of the injection, patients discussed the biosimilar with a healthcare professional. If they agreed to proceed, a new consent form was completed after the potential risks were explained, including stroke, myocardial infarction, and visual loss due to infection [8]. The intravitreal injection with the biosimilar was then administered.

Following the switch, patients continued to receive subsequent Ongavia injections based on disease activity and clinical requirements, following the same departmental protocol as used for the original molecule. For wet AMD, a Treat-and-Extend regimen was followed, in which the treatment interval was shortened or extended by two weeks depending on the presence or absence of intraretinal or subretinal fluid, until an interval of 12 weeks was achieved [9]. Once patients reached a stable 12-week interval, they received three consecutive 12-weekly injections before being shifted to a pro re nata (PRN) regimen. For macular oedema secondary to DMO and RVO, a PRN or as-required protocol was followed.

As the biosimilar switch was already underway in our trust, we conducted a study to evaluate patient satisfaction with the switching process and to assess the effectiveness of this transition.

Methods

The study was conducted in the Medical Retina Department of the Birmingham and Midland Eye Centre, Sandwell and West Birmingham NHS Trust.

The study had two components. The first component was a cross-sectional observational survey, in which patients completed a satisfaction questionnaire. The questionnaire, shown in the Appendices, was designed to assess whether the information provided in the leaflet posted to patients’ home addresses regarding the switch from ranibizumab (Lucentis) to the biosimilar ranibizumab (Ongavia) was adequate, thorough, and supported informed decision-making.

All decisions regarding the switch to biosimilar ranibizumab were made under the advice and supervision of a Medical Retina Consultant. On the day of the injection, after discussing the biosimilar with the clinician, the questionnaire was distributed to patients before they received their injections.

Following the switch, the second part of the study aimed to assess the clinical effectiveness and safety of the biosimilar ranibizumab (Ongavia) in patients with wet AMD, and its impact on patients’ treatment intervals.

Six months after the switch, we retrospectively reviewed all patients who had been switched to Ongavia. However, for the analysis, only wet AMD cases were considered. For the second component, the inclusion criteria consisted of patients with wet AMD who were switched to Ongavia, managed under the treat-and-extend protocol, and who had received at least two Ongavia injections.

For each patient, we compared the last treatment interval (in weeks) while receiving the original ranibizumab injections with the treatment interval (in weeks) planned after the second Ongavia injection. Pre-switch and post-switch optical coherence tomography (OCT) scans were also reviewed to determine the presence or absence of intraretinal and/or subretinal fluid, as an indicator of disease activity following the switch.

Data collection and analysis

The data were collected and analysed using Microsoft Excel (Microsoft Corporation, Redmond, Washington). In the first component, questionnaire responses were obtained using a paper-based form, and all variables were entered into Microsoft Excel. Descriptive statistics were used, and results were expressed as percentages.

In the second component, the variables of interest were the treatment intervals (in weeks) before the switch and after two Ongavia injections, to determine whether the treatment intervals remained the same, were extended, or were shortened post-switch. In addition, the presence or absence of intraretinal and/or subretinal fluid after two Ongavia injections, and any adverse events, were also noted. Descriptive statistics were used to analyse treatment intervals, the presence or absence of fluid, and adverse events, and results were expressed as percentages.

## Results

The results of this study included data collection over three months, from November 15, 2023, to February 15, 2024.

During the study period, 138 eyes of 107 patients were receiving intravitreal ranibizumab injections. Of these 138 eyes, 17 (12.3%) completed their treatment as per protocol, leaving 121 eyes on ranibizumab treatment.

Out of the 121 eyes, 92 (76%) were switched to the ranibizumab biosimilar, Ongavia, 18 (15%) continued on ranibizumab, and ranibizumab treatment was discontinued in 11 eyes (9%). There were multiple reasons for discontinuation. Five eyes were switched to a different anti-VEGF agent due to an insufficient response to ranibizumab. Four eyes had only one injection remaining to complete their treatment, as per the treat-and-extend protocol. Treatment was discontinued in two eyes due to patient preference. 

First-component results

Out of 107 patients, 64 (60.7%) completed the questionnaire, which was administered following a discussion about the biosimilar ranibizumab (Ongavia).

**Figure 1:**
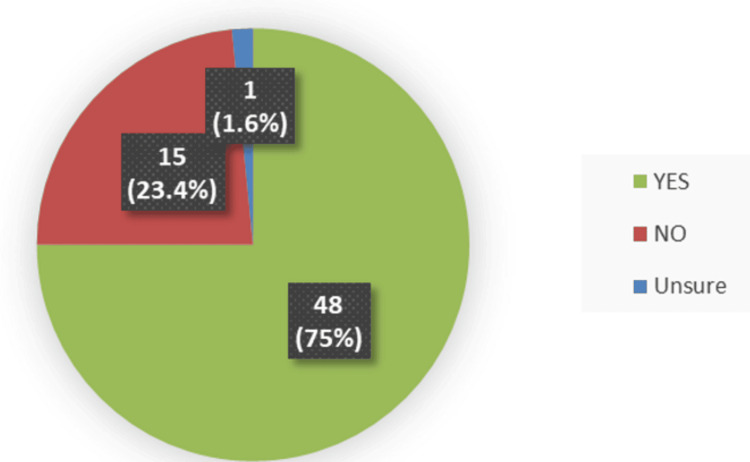
Patients’ questionnaire responses regarding receipt of the information leaflet at their home address (n = 64). Forty-eight patients (75%) reported receiving the leaflet, 15 (23.4%) reported not receiving it, and one (1.6%) was unsure.

Among the 64 patients who completed the satisfaction questionnaire, 48 (75%) had already received an information leaflet about the biosimilar ranibizumab (Ongavia) at their home address (Figure 1).

**Figure 2:**
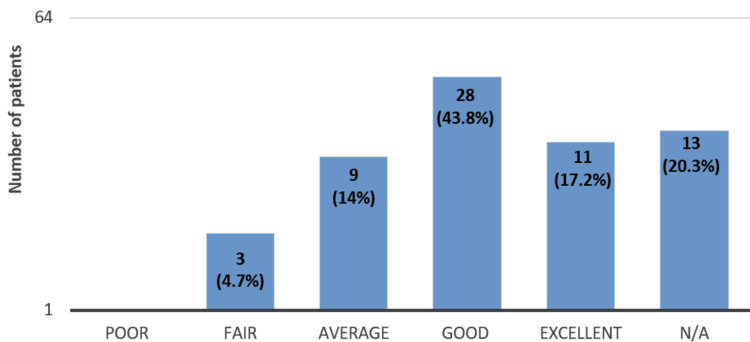
Patients’ questionnaire responses regarding how informative the Ongavia leaflet was about the biosimilar ranibizumab (n = 64). Twenty-eight patients (43.8%) rated the information as good, and 11 (17.2%) as excellent. Thirteen (20.3%) of the 64 patients did not answer the above question, while 3 (4.7%) of the patients found the information provided to be fair.

Thirty-nine (60.9%) of the 64 patients rated the information mentioned in the leaflet as above average (Figure 2).

**Figure 3:**
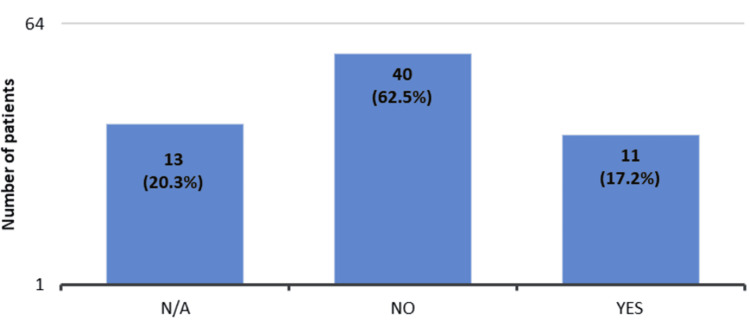
Patients’ questionnaire responses regarding whether they had questions after receiving the information leaflet about the biosimilar ranibizumab (Ongavia) (n = 64). Forty (62.5%) of the 64 patients did not have any questions after reading the information leaflet. Thirteen (20.3%) of the 64 patients did not answer this question when they were filling out the questionnaire, and 11 (17.2%) had some questions related to the biosimilar ranibizumab (Ongavia).

**Figure 4:**
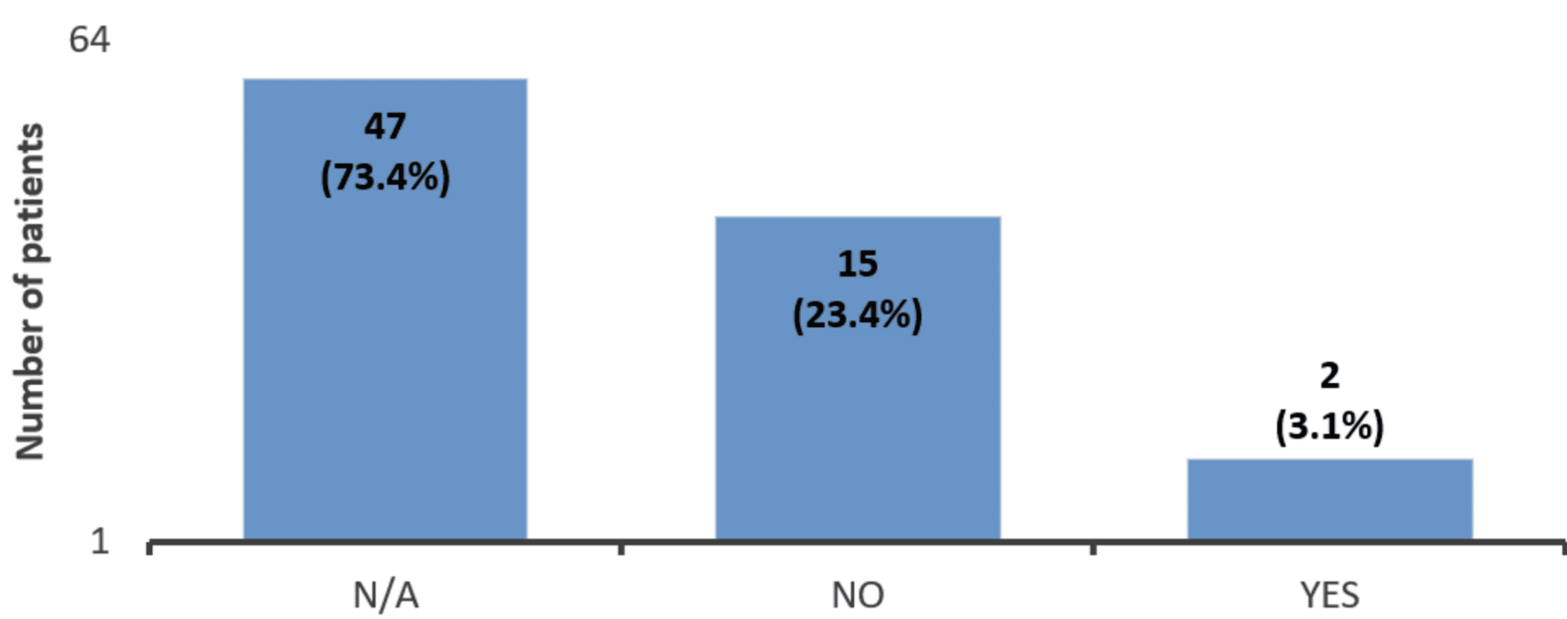
Patients’ questionnaire responses regarding whether they contacted the clinic to obtain further information about the biosimilar ranibizumab (Ongavia) (n = 64). Forty-seven (73.4%) of the 64 patients did not provide an answer to this question. Two (3.1%) of the 64 patients called us to learn more about the biosimilar ranibizumab.

Eleven (17.2%) of the 64 patients had questions after reading the leaflet (Figure 3), and 2 (3.1%) patients were contacted again to learn more about the medication (Figure 4).

**Figure 5:**
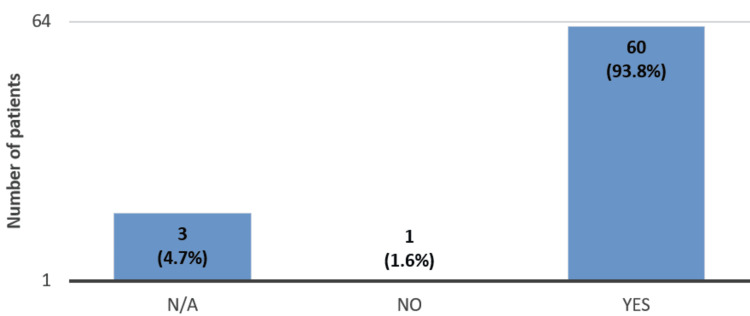
Patients’ questionnaire responses regarding whether the face-to-face discussion addressed their questions about the biosimilar ranibizumab (Ongavia) (n = 64). Sixty (93.8%) of the 64 patients had all the questions answered during the face-to-face discussion. Three (4.7%) of the 64 patients did not answer the above question while they filled out the questionnaire, and only one (1.6%) did not have all the questions answered.

During the face-to-face discussion, 60 (93.8%) of the 64 patients had all their questions answered (Figure 5).

Overall, 64 out of 64 patients who completed the questionnaire expressed satisfaction with the entire information process. 

Second component: treatment intervals and OCT results

In the second part, we retrospectively reviewed the OCT scans and treatment intervals of 92 eyes that were switched to the ranibizumab biosimilar, Ongavia.

Out of the 92 eyes, 90 eyes of patients with wet AMD were considered. One eye received treatment for DMO and one for cystoid macular oedema (CMO) secondary to retinal vein occlusion, and these were not included in the second part of the study.

Of these 90 eyes with wet AMD, three eyes of two patients were excluded, as one patient’s treatment was paused due to a heart attack, and one patient, who was receiving treatment in both eyes, had died.

A retrospective review conducted on 87 eyes with wet AMD receiving Ongavia injections, in accordance with the treat-and-extend protocol, revealed the following outcomes.

**Figure 6:**
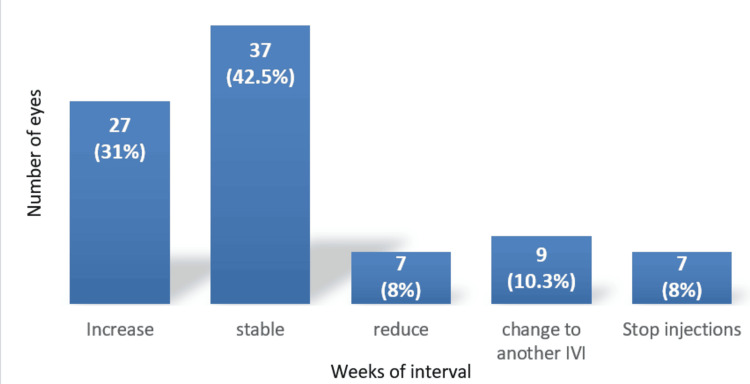
Effect of the biosimilar ranibizumab (Ongavia) on treatment interval duration under the treat-and-extend (TREX) regimen (n = 87 eyes). Sixty-four (73.5%) of the 87 eyes increased or maintained the treatment interval with the biosimilar Ongavia injections, while 7 (8%) of the 87 eyes reduced it.

Out of the 87 eyes, in 64 eyes (73.5%), the treatment interval (in weeks) was either increased or maintained. The treatment interval was reduced in seven eyes (8%). Nine eyes (10.3%) were switched to another anti-VEGF medication due to poor response to Ongavia injections. Seven eyes (8%) completed the treat-and-extend protocol and treatment was finished (Figure 6).

Furthermore, the treatment interval was reduced in seven (8%) out of 87 eyes due to a combination of sub-optimal response to Ongavia and disease reactivation.

Nine (10.3%) out of 87 eyes were switched to another anti-VEGF medication. Of these nine eyes, six (66.6%) had shown sub-optimal responses to multiple ranibizumab injections previously, and the response remained sub-optimal with Ongavia. In two cases, the switch occurred after patients missed a follow-up injection with Ongavia, prompting the clinician to change the medication. Only one eye (11.1%) out of nine eyes developed fluid after switching to Ongavia, whereas there had been no fluid on OCT while receiving ranibizumab. This case was switched to another anti-VEGF medication.

Of the 18 eyes that were initially continued on ranibizumab injections, seven eyes (39%) were maintained on ranibizumab, four eyes (22.2%) completed their treat-and-extend treatment with ranibizumab, two eyes (11.1%) were switched to the biosimilar Ongavia at a later stage, and four eyes (22.2%) were switched to another anti-VEGF drug, three eyes to faricimab and one eye to aflibercept. In one eye (5.5%), treatment was discontinued due to the patient’s choice.

Regarding side effects, all patients’ clinical notes were reviewed retrospectively. No ocular side effects were documented in the clinical notes.

## Discussion

This study highlights the real-world results of the first biosimilar medication switch in ophthalmology patients at the Birmingham and Midland Eye Centre.

Overall, patient satisfaction with the information process was high. Questionnaire responses showed that the majority of patients had all their questions answered during the face-to-face discussion, indicating that patients felt confident proceeding with the biosimilar Ongavia. Furthermore, leaflet distribution containing the necessary information about biosimilar medication, together with a detailed face-to-face discussion, demonstrates that clear communication and shared decision-making facilitated patient acceptance of switching to a biosimilar medication.

Although most patients reported that their questions about the biosimilar were answered during the consultation, only one patient felt that not all their questions were addressed. This highlights the importance of direct and clear communication in addressing concerns about biosimilar medications. Actively listening to patient concerns and involving them in treatment decisions contributed significantly to the high satisfaction rate [10].

The clinical effectiveness of the biosimilar ranibizumab was evaluated by monitoring injection intervals for patients treated for wet AMD and by reviewing OCT scans. The results showed that injection intervals were either maintained or increased, with stable OCT findings, in the majority of eyes (73.5%) after receiving Ongavia. Maintaining or increasing injection intervals is clinically meaningful, as patients require less frequent injections, indicating that Ongavia is comparable to ranibizumab.

This study suggests that the clinical effectiveness of biosimilar ranibizumab (Ongavia) is similar to ranibizumab, with results comparable to other published studies [11]. Chalkiadaki et al. (2024) demonstrated no statistically significant difference in injection intervals or the presence of fluid in patients switched to the biosimilar Ongavia. In addition, Sharma et al. (2024) compared the effectiveness of a different biosimilar ranibizumab, Razumab, with ranibizumab in treatment-naïve wet age-related macular degeneration patients, and reported similar improvements in vision and resolution of fluid on OCT scans, with comparable safety [12]. Sharma et al. (2025), in a multicentric retrospective study of 1230 patients receiving 3595 ranibizumab biosimilar (FYB 201) injections for various medical retina conditions, reported stable mean best corrected visual acuity (BCVA) and a significant reduction in central foveal thickness. Adverse effects were minimal, with three eyes (0.24%) experiencing ocular adverse effects and six patients (0.48%) experiencing systemic adverse effects, supporting the biosimilar’s safety and efficacy [13].

Adverse events documented in the present study were consistent with those reported in other studies [11,14]. During the study, no ocular adverse effects were observed with the biosimilar Ongavia. One patient experienced a myocardial infarction following the first intravitreal injection of Ongavia. Cardiovascular events are recognised potential risks associated with anti-VEGF injections [8]; however, a causal relationship cannot be established from this single observation. Additionally, one patient receiving treatment in both eyes died due to unrelated causes.

The limitations of this study include its retrospective design, the absence of a control group, and the short duration of follow-up. The clinical effectiveness of biosimilar Ongavia was evaluated by assessing disease activity based on the presence or absence of fluid on OCT scans and clinicians’ decisions to increase or decrease treatment intervals. Changes in visual acuity were not assessed, and central macular thickness was not measured or compared.

This study demonstrates that, for a smooth transition from an originator medication to its biosimilar, patients must be actively involved in decision-making regarding their treatment options. Changes in treatment should continue to involve senior clinicians, particularly during the re-consenting process. No new safety concerns were identified with the biosimilar ranibizumab (Ongavia), and it was clinically comparable to ranibizumab. Use of this biosimilar could represent a cost-effective approach to delivering high-quality patient care.

## Conclusions

While the patient information process contributed to high acceptance and satisfaction rates, the granular details regarding leaflet distribution may not be central to the main objectives of the study. The broader relevance of our findings lies in demonstrating that biosimilar ranibizumab (Ongavia) provides comparable clinical effectiveness to the reference product, supporting its role as a cost-conscious alternative in the long-term management of wet AMD. By confirming similar injection intervals and disease activity profiles post-switch, our results contribute to the growing body of evidence promoting biosimilars as a strategy to alleviate economic burden without compromising patient outcomes.

## Figures and Tables

**Figure 7:**
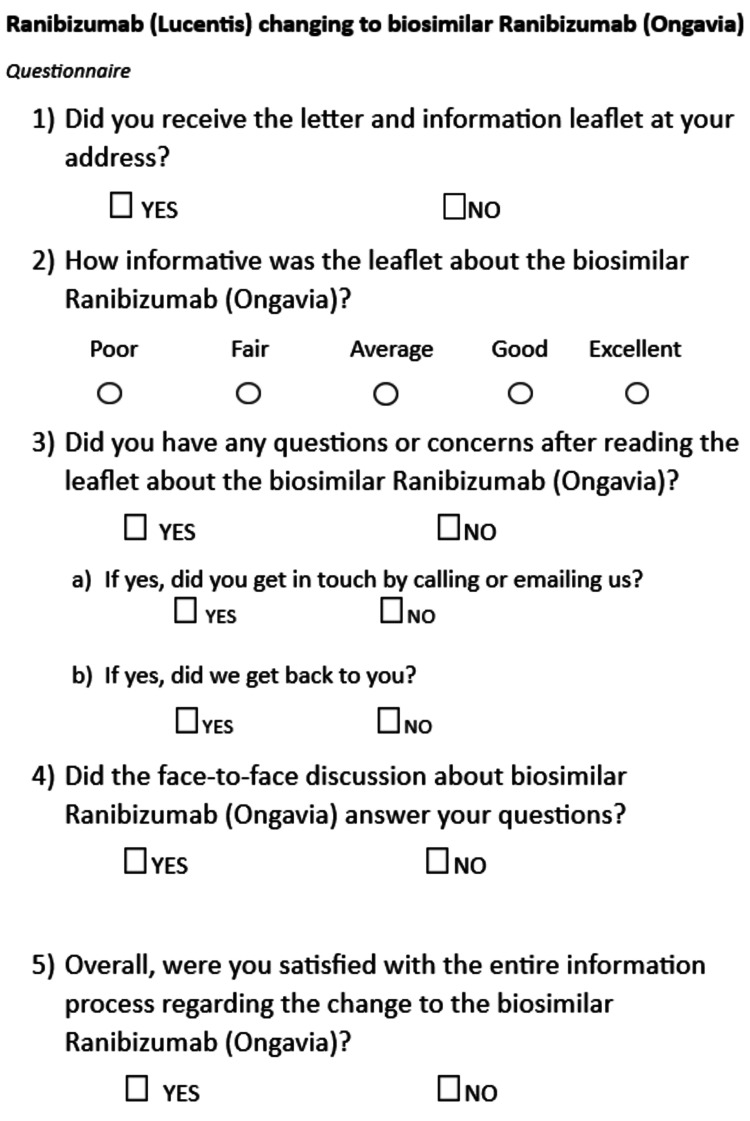
The satisfactory questionnaire. This questionnaire was given to the patients to complete at the end of the face-to-face discussion about the biosimilar ranibizumab (Ongavia).
